# Venous Thromboembolism in Patients Hospitalized for COVID-19 in a Non-Intensive Care Unit

**DOI:** 10.3390/jcm13020528

**Published:** 2024-01-17

**Authors:** Magdalena Mackiewicz-Milewska, Małgorzata Cisowska-Adamiak, Jerzy Pyskir, Iwona Świątkiewicz

**Affiliations:** 1 Department of Rehabilitation, Nicolaus Copernicus University in Toruń, Collegium Medicum in Bydgoszcz, 85-094 Bydgoszcz, Poland; malgorzata.cisowska@cm.umk.pl; 2 Department of Biophysics, Nicolaus Copernicus University in Toruń, Collegium Medicum in Bydgoszcz, 85-094 Bydgoszcz, Poland; jerzy_pyskir@cm.umk.pl; 3 Division of Cardiovascular Medicine, University of California San Diego, La Jolla, CA 92037, USA; iwona.swiatkiewicz@gmail.com

**Keywords:** COVID-19, SARS-CoV-2 variant, venous thromboembolism, pulmonary embolism, deep vein thrombosis, D-dimer, thromboprophylaxis

## Abstract

Coronavirus Disease 2019 (COVID-19) caused by Severe Acute Respiratory Syndrome Coronavirus 2 (SARS-CoV-2) may contribute to venous thromboembolism (VTE) with adverse effects on the course of COVID-19. The purpose of this study was to investigate an incidence and risk factors for VTE in patients hospitalized for COVID-19 in a non-intensive care unit (non-ICU). Consecutive adult patients with COVID-19 hospitalized from November 2021 to March 2022 in the isolation non-ICU at our center were included in the study. Incidence of VTE including pulmonary embolism (PE) and deep vein thrombosis (DVT), clinical characteristics, and D-dimer plasma levels during the hospitalization were retrospectively evaluated. Among the 181 patients (aged 68.8 ± 16.2 years, 44% females, 39% Delta SARS-CoV-2 variant, 61% Omicron SARS-CoV-2 variant), VTE occurred in 29 patients (VTE group, 16% of the entire cohort). Of them, PE and DVT were diagnosed in 15 (8.3% of the entire cohort) and 14 (7.7%) patients, respectively. No significant differences in clinical characteristics were observed between the VTE and non-VTE groups. On admission, median D-dimer was elevated in both groups, more for VTE group (1549 ng/mL in VTE vs. 1111 ng/mL in non-VTE, *p* = 0.09). Median maximum D-dimer was higher in the VTE than in the non-VTE group (5724 ng/mL vs. 2200 ng/mL, *p* < 0.005). In the univariate analysis, systemic arterial hypertension and the need for oxygen therapy were predictors of VTE during hospitalization for COVID-19 (odds ratio 2.59 and 2.43, respectively, *p* < 0.05). No significant associations were found between VTE risk and other analyzed factors; however, VTE was more likely to occur in patients with a history of VTE, neurological disorders, chronic pulmonary or kidney disease, atrial fibrillation, obesity, and Delta variant infection. Thromboprophylaxis (83.4% of the entire cohort) and anticoagulant treatment (16.6%) were not associated with a decreased VTE risk. The incidence of VTE in patients hospitalized in non-ICU for COVID-19 was high despite the common use of thromboprophylaxis or anticoagulant treatment. A diagnosis of arterial hypertension and the need for oxygen therapy were associated with an increased VTE risk. Continuous D-dimer monitoring is required for the early detection of VTE.

## 1. Introduction

Coronavirus Disease 2019 (COVID-19) caused by Severe Acute Respiratory Syndrome Coronavirus 2 (SARS-CoV-2) was an extraordinary challenge across the globe between 2019 and 2022, which led to excess morbidity and millions of deaths [1,2]. By January 2022, more than 310 million people had been infected worldwide, with 6.3 million deaths as of June 2022 [1,2].

The main symptoms of the disease are fever, cough, muscle pain, and shortness of breath. While respiratory complications are mainly responsible for the severe course of COVID-19, often requiring hospitalization in an intensive care unit (ICU), complications related to venous thromboembolism (VTE) including pulmonary embolism (PE) and deep vein thrombosis (DVT) are equally serious. It has not yet been clearly determined what group of patients with COVID-19 is at greater risk of VTE complications, especially among those who are hospitalized in a non-ICU [3,4,5,6]. The reported incidence of VTE during COVID-19 varies considerably, ranging from 6 to 85% [3,4,5,7,8,9]. The large variability of VTE incidence in patients hospitalized for COVID-19 may be related to differences in patient sampling, hospital setting, the presence of symptoms, and diagnostic protocols for VTE detection [3,4,5,6,7,8,9,10,11,12]. The overall incidence of VTE in patients hospitalized for COVID-19 has been reported to range from 7% to 26% [3,4,8,9]. VTE occurred more frequently in patients admitted to ICU (9–85% of patients) compared to those hospitalized in non-ICU (6–27%) [4,5,6,7,8,9]. Among patients hospitalized in the ICU, VTE incidence was 9% in [12], 20% in [6], 24% in [4,9], 31% in [10], 46% in [8], 69% in [11], 76% in [5], and 85% in [4]. In patients hospitalized in a non-ICU, VTE incidence was 6% in [6], 8% in [9], 9% in [4], 12% in [7], 23% in [8], and 27% in [5]. The relative risk for VTE associated with ICU admission was reported to be as high as 2.99 [9]. It should be noted that applying systematic screening for VTE, mainly using the ultrasound examination of veins, resulted in an increase in VTE rates compared to an approach based on the clinical suspicion of VTE both in ICU and non-ICU patients [4,8]. In the study of Avruscio et al. [5], in which all patients underwent a systematic ultrasound screening of veins, VTE was diagnosed in 27% of patients hospitalized in non-ICU and 76% of those admitted to ICU. In the meta-analysis of 91 studies performed by Mansory et al. [9], VTE incidence was 12% and 19% in the general population of hospitalized COVID-19 patients, 21% and 34% of ICU patients, and 6% and 11% of non-ICU patients, depending on whether screening was symptom-triggered or mandatory. Prolonged immobilization or restricted mobility, a need for oxygen therapy, and a prolonged bedridden state due to a severe course of COVID-19 itself [7], as well as a COVID-19-related dysregulation of the coagulation cascade and intravascular coagulopathy are among important factors that may contribute to an occurrence of VTE [13].

The purpose of this study was to investigate the incidence and risk factors for VTE in patients hospitalized for COVID-19 in a non-ICU.

## 2. Materials and Methods

### 2.1. Study Design and Patients

We performed a retrospective longitudinal cohort study of patients with a diagnosis of COVID-19 hospitalized in the non-ICU at the University Hospital No. 1 in Bydgoszcz, Poland, from 17 November 2021 to 30 March 2022. It is important to note that based on the Polish national database on COVID-19 for the period from 1 March 2020 through 30 June 2022, the most severe course of COVID-19 with the highest mortality, a frequent need for oxygen therapy and mechanical ventilation, and the longest hospitalizations was observed from 1 July through 31 December 2021 which is defined as a Delta period [14]. Then, the Omicron period (1 January–30 June 2022) was characterized by a better outcome. Our study started in November 2021 and therefore included both the Delta and Omicron periods.

Patients were admitted to the isolation Ward No. 5 at our center which had been temporarily transformed from the Department of Rehabilitation. The ward consisted of 25 beds, including 4 beds with a possibility to perform a mechanical ventilation of the lungs. Patients were admitted to the isolation non-ICU either from the hospital emergency ward or from other hospital wards if they were diagnosed with COVID-19 requiring hospitalization but not requiring admission to ICU [15]. Typically, patients with stage 2 or stage 3 of COVID-19 were admitted to the isolation ward at our center [15,16,17]. Patients > 60 years old and/or patients with obesity, diabetes mellitus, cancer, chronic heart failure, chronic respiratory failure, chronic renal failure, immunodeficiency, and immunosuppression, who were considered to be at risk of a severe course of COVID-19, were more likely to require hospitalization for COVID-19 [15]. Patients aged ≥18 years hospitalized for COVID-19 in the non-ICU at our center who had available medical records to review were eligible for this study.

Hospital medical records were retrospectively reviewed for collecting data on clinical characteristics, initial and maximum D-dimer plasma levels, and the incidence of VTE during hospitalization for COVID-19. VTE was defined as an occurrence of PE, DVT, or a combination of both. Associations between clinical characteristics and VTE occurrence were analyzed. The following variables were included in this analysis: age, gender, the variant of SARS-CoV-2 (Delta or Omicron), obesity, smoking, a history of VTE, a need for oxygen therapy, the type of oxygen therapy that was administered, the use of thromboprophylaxis or anticoagulant treatment, and comorbidities (such as systemic arterial hypertension, coronary artery disease, diabetes, chronic heart failure, atrial fibrillation, chronic kidney disease, chronic pulmonary disease, neurological disorders, and cancer). Data on the comorbidities and past medical history were acquired through the medical record review. Coronary artery disease was diagnosed based on the data on previous myocardial infarction or unstable angina, ≥50% stenosis of a major coronary artery in coronary angiography, and intracoronary interventions in past medical history. A diagnosis of systemic arterial hypertension was made based on the patients’ medical records or the current use of antihypertensive medication(s). A diagnosis of diabetes was made based on the patients’ medical records or the current use of antidiabetic medication(s). Chronic kidney disease referred to the presence of stage 3 or a more severe stage of the disease. Chronic pulmonary disease was identified based on the diagnosis of chronic obstructive pulmonary disease, emphysema, bronchial asthma, and chronic vascular pulmonary disease. Neurological disorders included previous stroke, sclerosis multiplex, Parkinson’s disease, vascular brain damage, or dementia. The diagnosis of cancer (previous or current) was made on the basis of medical record data. A history of VTE was indicated by evidence of previous PE and/or DVT based on patient medical records. Obesity was diagnosed if the body mass index was above 30 kg/m^2^.

The study was conducted in accordance with the Declaration of Helsinki. Approval No. 497/2022 was obtained from the Bioethics Committee of the Collegium Medicum in Bydgoszcz, Nicolaus Copernicus University in Toruń, to carry out the retrospective study. Written informed consent for participation in the study was waived given the retrospective nature of this analysis.

### 2.2. Diagnostic Tests and Patient Management

RT-PCR test (real time reverse transcription–polymerase chain reaction test) was performed in all patients to confirm the SARS-CoV-2 infection causing COVID-19. Based on the national database including data on the SARS-CoV-2 variants during the COVID-19 pandemic in Poland, we assumed that during our study, the Delta SARS-CoV-2 variant was predominant in the period from 17 November 2021 to 31 December 2021 and the Omicron variant from 1 January 2022 to 30 March 2022 [14].

On the day of hospital admission, all patients underwent high resolution computed tomography (HRCT) of the lungs to investigate COVID-19-related inflammatory changes.

A chest CT angiogram (CTA) was performed on admission in patients with a clinical suspicion of PE, severe dyspnea, and in those whose D-dimer plasma level was above 2500 ng/mL. During hospitalization, a chest CTA was performed to exclude PE in patients with a sudden increase in D-dimers (monitored daily), increasing dyspnea, sudden decrease in oxygen saturation (SpO_2_) (especially when SpO_2_ dropped below 94%), or other symptoms leading to clinical suspicion of PE. PE was diagnosed if embolic material was found in the pulmonary arteries in the CTA scan.

DVT of the lower and upper extremities was diagnosed using compression and Doppler study (CDUS). The examination was performed in patients who experienced a swelling, pain, redness, or increased warmth of the limb. An abnormal study was characterized by a dilated incompressible vessel, with the presence of low-level intravascular echoes indicating a thrombus and an absent or continuous Doppler velocity signal.

D-dimer plasma levels were routinely measured upon hospital admission and then monitored daily for 7 days. Thereafter, D-dimer measurements were made if needed, e.g., on clinical suspicion of DVT or PE. Plasma levels of D-dimer were determined using HemosIL D-Dimer HS 500, an automated latex enhanced immunoassay (Instrumentation Laboratory IL). D-dimer plasma concentration was considered within normal reference range when D-dimer was ≤500 ng/mL.

Patients received specific basic and supportive treatment for COVID-19 according to the national guidelines on managing COVID-19 patients and depending on symptoms and the severity of the disease [15,16,17].

Low-molecular-weight heparin (LMWH) in a standard prophylactic dose, i.e., enoxaparin 40 mg once daily, was administered to all patients [15,16,17,18]. Patients who were receiving chronic oral anticoagulant treatment at the time of admission for various reasons, e.g., atrial fibrillation or a history of VTE, were discontinued from oral anticoagulants and given enoxaparin in a therapeutic dose, i.e., 1 mg/kg every 12 h based on total body weight.

An assessment of hypoxia severity was performed through measurements of oxygen saturation (SpO_2_): continuously in patients on high-flow nasal cannula (HFNC) oxygen therapy, non-invasive ventilation (NIV), and invasive mechanical ventilation; every two hours in patients on passive oxygen therapy; and four times a day in stable patients not requiring oxygen therapy. In patients with severe inflammatory changes in the lungs and ineffective passive oxygen therapy, a monitoring of arterial blood gas test was performed. Dependently on the severity of hypoxia and signs of respiratory failure, the following types of oxygen therapy were used: (1) passive oxygen therapy in the form of oxygen nasal cannula (oxygen flow rate up to 6 L/min) for mild hypoxia, (2) passive oxygen therapy in the form of face mask (oxygen flow rate of 10–20 L/min) for mild hypoxia, (3) high-flow nasal cannula (HFNC) oxygen therapy (oxygen flow rate up to 70 L/min) for acute respiratory failure, (4) non-invasive ventilation (NIV) with high-flow oxygen equipment, if the use of HFNC was ineffective, and (5) invasive mechanical ventilation, if NIV was ineffective. Patients receiving invasive or non-invasive ventilation were transferred to the ICU, but if there were no vacancies there, patients were treated in the non-ICU.

### 2.3. Outcomes

The main outcome was an occurrence of VTE that was defined as an occurrence of pulmonary embolism (PE), deep vein thrombosis (DVT), or the combination of both during the hospitalization for COVID-19 in the non-ICU.

Further, an exploratory analysis of differences in clinical characteristics including demographic variables, comorbidities, SARS-CoV-2 variant (Delta or Omicron), D-dimer plasma levels, and the use of thromboprophylaxis, anticoagulant treatment, and oxygen therapy between groups of patients experiencing VTE (VTE group) and those who did not experience VTE (non-VTE group) was conducted. We analyzed collected data to determine risk factors for VTE in the COVID-19 patients hospitalized in the non-ICU.

### 2.4. Statistical Analysis

In order to select a test to investigate differences between samples, the normality of the distributions of the measured parameters was examined using the Shapiro–Wilk test. Since some of the parameters did not have a normal distribution, the statistical significance of the differences was determined using the Mann–Whitney U test between independent samples. In order to determine the odds ratio (OR) of the occurrence of VTE complications depending on various factors, the univariate logistic regression model was used. The univariate analysis includes clinical variables including a history of VTE, obesity, smoking, and a diagnosis of comorbidities such as arterial hypertension, coronary artery disease, diabetes, chronic heart failure, atrial fibrillation, cancer (previous or current), chronic kidney disease, chronic pulmonary disease, neurological disorders, as well as SARS-CoV-2 variant (Delta or Omicron), the use of thromboprophylaxis or anticoagulant treatment, and a need for oxygen therapy. The results of statistical tests were accepted as statistically significant when *p* < 0.05.

## 3. Results

A total of 181 consecutive adult patients hospitalized in the non-ICU due to COVID-19 were included in the study. The SARS-CoV-2 infection causing COVID-19 was confirmed by means of the RT-PCR test in all patients. Of this group, 18 patients (i.e., 9.9%) died during the hospitalization for COVID-19 and 14 patients (i.e., 7.7%) required transfer to the ICU due to symptomatic worsening, mainly hypoxemic respiratory failure/acute respiratory distress syndrome (ARDS). The mean length of hospitalization for COVID-19 in the non-ICU was 13.5 ± 6.7 days. All patients who developed VTE during hospitalization were administered anticoagulant treatment with the LWMH, i.e., enoxaparin 1 mg/kg of total body weight every 12 h.

The older age of the studied population and the high prevalence of cardiovascular disease such as arterial hypertension (~62% of the entire cohort), coronary artery disease (~26%), and chronic heart failure (~21%), as well as the frequent occurrence of diabetes (~35%), obesity (~21%), cancer (~20%), chronic kidney disease (~18%), and a history of VTE (~17%) indicate an increased risk of a severe course of COVID-19 in the patients included in our study (Table 1) [10]. Most patients were hospitalized for COVID-19 in the period characterized by the predominance of the Omicron SARS-CoV-2 variant (~61% of the entire cohort). Notably, all patients in both groups received thromboprophylaxis on hospital admission according to the national guidelines on managing COVID-19 patients [15,16,17], unless they required anticoagulant treatment for various reasons (Table 1). The majority of patients included in our study received oxygen therapy (~60% of the entire cohort).

Among the 181 patients included in our study, VTE in the form of PE and DVT was diagnosed during hospitalization for COVID-19 in 29 patients, representing 16% of the entire study population. Specifically, PE was diagnosed in 15 patients (8.3% of the entire cohort) and DVT in 14 patients (7.7%). PE events were not associated with DVT. While DVT of the lower limbs was diagnosed in 11 patients (DVT of the proximal veins in 7 patients, DVT of the distal veins in 2 patients, and DVT of both the proximal and distal veins in 2 patients), 3 patients had DVT of the upper limbs. The remaining 152 patients were not diagnosed with VTE during the hospitalization for COVID-19. The patients with VTE and without VTE are denoted as VTE group and non-VTE group, respectively.

No significant difference was observed between the VTE group and the non-VTE group in the length of hospitalization for COVID-19 (13.1 ± 5.1 days vs. 13.6 ± 7.0 days, *p* = 0.99). During the hospitalization for COVID-19, 3 patients from the VTE group (10.3% of the group) and 15 patients from the non-VTE group (9.9% of the group) died (*p* between groups = 0.7). Among the three patients with VTE who died, two had PE and lung lesions typical of COVID-19 occupying more than 50% of the lungs. A need for transfer to ICU due to symptomatic worsening was 2-fold more frequent in patients from the VTE group (4 patients, 13.8% of the group) compared to the non-VTE group (10 patients, 6.6% of the group; *p* between groups = 0.13). Of the four patients with VTE transferred to the ICU, two patients had PE and three had severe inflammatory changes in the lungs typical of COVID-19 occupying more than 50% of the lungs.

The comparison of clinical characteristics during the hospitalization for COVID-19 for the groups of patients with and without VTE is shown in Table 1. No significant differences in clinical characteristics including the frequency of receiving thromboprophylaxis or anticoagulant treatment were found between the VTE group and non-VTE group; however, arterial hypertension, chronic pulmonary disease, and neurological disorders were more likely to occur in the VTE group. Moreover, while ~17% of patients from the VTE group had a history of VTE, no VTE history was found in patients from the non-VTE group. Although no significant difference was observed between the VTE and non-VTE group in the need for oxygen therapy during hospitalization for COVID-19, patients from the VTE group were more likely to require this type of treatment. While the Omicron SARS-CoV-2 variant was a dominant etiologic factor for COVID-19 in patients from the non-VTE group, there was no predominance of any SARS-CoV-2 variant in patients with VTE complications.

The results of D-dimer plasma level measurements in the groups of patients with and without VTE are displayed in Table 2. The VTE-group and non-VTE group did not differ in D-dimer levels on hospital admission (*p* = 0.09); however, initial D-dimer was more likely to be higher in the VTE group. Median maximum D-dimer level was significantly higher in the VTE group compared to the non-VTE group (5724 ng/mL vs. 2200 ng/mL, *p* < 0.005).

In a univariate analysis, patients with a diagnosis of arterial hypertension and using oxygen therapy had a significantly higher risk of VTE complications during hospitalization for COVID-19. No significant associations were found between VTE and other analyzed factors; however, VTE was more likely to occur in patients with a history of VTE, neurological disorders, chronic pulmonary disease, atrial fibrillation, chronic kidney disease, obesity, and a Delta SARS-CoV-2 variant infection. Thromboprophylaxis and anticoagulant treatment were not associated with a decreased VTE risk. The lack of statistical significance of these associations may result from the relatively small size of the analyzed groups. The results of the univariate logistic regression analysis are shown in Table 3.

The risk of VTE complications in the course of COVID-19 among the patients hospitalized in the non-ICU depending on the type of oxygen therapy is shown in Table 4. The majority of patients receiving oxygen therapy in both the VTE and non-VTE group obtained passive oxygen therapy; however, about one fifth of patients from the VTE group required mechanical ventilation. While there were no statistically significant differences between the VTE and non-VTE group in the types of administered oxygen therapy, patients with VTE were most likely to require oxygen therapy, especially high-flow nasal cannula oxygenation (HFNC).

## 4. Discussion

COVID-19 induced by SARS-CoV-2 can lead to an increased risk of VTE that may contribute to a more severe course of the disease with adverse outcome and increased mortality. Numerous studies provided evidence that the following factors may be responsible for VTE complications in the course of COVID-19: vascular endothelial cell dysfunction, hyper-inflammatory immune response, platelet activation, blood stasis, and COVID-19-associated coagulopathy (CAC) [19,20,21]. CAC manifests as micro- and macro-arterial and venous thromboses that can affect multiple organs including the lungs, heart, brain, and kidneys [21]. CAC is associated with an elevation in D-dimer levels which is observed in many COVID-19 patients [19,22]. Among the VTE complications in the course of COVID-19, PE and DVT are the most common. The incidence of VTE in patients with COVID-19 is estimated to be between 6% and about 40% (while autopsies raise it to about 60%) and tends to increase in hospitalized patients, especially those critically ill who are treated in the ICUs [3,4,5,6,7,8,9,10,11,12,21,23,24,25,26,27]. Additionally, patients with COVID-19 and VTE compared to those without VTE had a higher risk of death [5,6,28].

The findings of our study emphasize the significance of VTE risk in patients hospitalized for COVID-19 who did not require ICU treatment. The incidence of VTE in this group of patients was high despite guideline-based therapies. A retrospective analysis of 181 patients hospitalized for COVID-19 in the non-ICU at our center between November 2021 and March 2022 showed that 16% of patients developed VTE complications in the form of PE and DVT. A significantly higher risk of VTE was observed in patients suffering from arterial hypertension and requiring oxygen therapy. While no significant associations were found between VTE and other analyzed factors, VTE was more likely to occur in patients with a history of VTE, neurological disorders, chronic pulmonary and kidney disease, atrial fibrillation, obesity, and a Delta SARS-CoV-2 variant infection. Notably, D-dimer plasma levels were significantly higher during hospitalization and were more likely to be elevated on hospital admission in patients with VTE compared to those without. In addition, thromboprophylaxis and anticoagulation treatment were not associated with a decreased VTE risk. Based on our findings, the course of COVID-19 was more severe in patients with VTE complications as there was an approximately 2-fold higher rate of transfer to ICU due to symptomatic worsening and a substantially more frequent need of oxygen therapy in this group compared to the group of patients without VTE.

In contrast to our findings, a meta-analysis of 21 studies including 5296 patients hospitalized for severe COVID-19 in ICU showed that arterial hypertension did not contribute to an increased risk of VTE [29]. Also, no significant associations between VTE risk and several clinical factors which can be considered as traditional VTE risk factors such as obesity, a history of VTE, immunodeficiency, and cancer were observed in that study. However, indices of COVID-19 severity (such as the length of hospitalization and a need for mechanical ventilation) were significantly associated with VTE risk [28]. Also, a significantly higher incidence of VTE complications was observed in ICU patients with COVID-19 than in patients hospitalized in the ICU for other reasons [21]. These findings suggest that factors related to COVID-19 itself such as a severe course of COVID-19 with hyperinflammation or CAC may have a greater impact on VTE development than traditional VTE risk factors. Our findings also indicate that more severe course of COVID-19 may contribute to a greater VTE risk as evidenced, for example, by the more frequent need for oxygen therapy in patients with VTE compared to patients without VTE. However, we also observed that other risk factors such as a diagnosis of arterial hypertension or a history of VTE may play an important role in developing VTE. In our study, patients experiencing VTE during hospitalization for COVID-19, but not patients from the non-VTE group, had a history of previous VTE incidents. The high prevalence of arterial hypertension in our cohort (62%) compared to other studies of COVID-19 patients (e.g., 50% in [8]) may contribute to a greater significance of hypertension for increasing VTE risk in our study. Notably, in the study of Poletto et al. [27] with a comparable hypertension prevalence (i.e., ~63% in entire cohort and 76% in the VTE group in [27] vs. 62% and 76%, respectively, in our study), regression analysis indicated that hypertension was likely to increase VTE risk (OR = 1.83, *p* = 0.08). There is also some evidence in the literature indicating that hypertension was associated with an ∼50% higher risk of VTE [30] and may be an important risk factor for DVT occurrence [31].

In their meta-analysis, Nopp et al. [8] found that VTE risk in patients hospitalized for COVID-19 in ICUs is high but it is also substantial in non-ICU hospitalized patients. While the overall incidence of VTE in the entire population of COVID-19 patients was 14.1%, in those hospitalized in ICUs, it was 22.7%, but in the subgroup of patients hospitalized in non-ICUs, it was 7.9%. When screening for DVT (through the ultrasound examination of deep veins of the upper and/or lower extremities) was performed, these numbers increased significantly, i.e., up to 45.6% in ICU patients and 23% in non-ICU patients. Moreover, a meta-analysis of 30 studies including patients hospitalized for COVID-19 showed a high overall incidence of VTE including PE with or without DVT and DVT alone (26%, 12%, and 14%, respectively), especially when the ultrasound screening of veins was performed (DVT was found in up to 85% of patients) [4]. However, where referral to imaging tests was based on clinical suspicion, VTE, PE, and DVT were diagnosed in 24%, 19%, and 7% of patients hospitalized in ICU, respectively, and in 9%, 4%, and 7% of patients admitted to general wards, respectively [4]. When all patients underwent ultrasound screening for DVT and a multidetector computed tomographic angiography in the case of suspected PE in the study of Avruscio et al. [5], VTE incidence (defined as any DVT and symptomatic PE) was higher (27% in patients admitted to the general medical ward and 76% in ICU patients) than in our study of non-ICU patients (16%). Importantly, Avruscio et al. [5] prospectively investigated DVT occurrence in COVID-19 patients in different clinical settings by performing a systematic ultrasonographic assessment for DVT every 7 days during the whole hospital stay. In general, the systematic surveillance of all patients hospitalized for COVID-19 is recommended as opposed to limiting the investigation to the symptomatic patients. It is notable that the rate of PE in our study is similar (8.3%) to the study of Avruscio et al. [5] (9.8%), although we investigated only non-ICU patients while Avruscio et al. included both ICU and non-ICU patients. This result may be related to a similar approach for performing imaging tests on hospital admission.

It should be noted that ultrasound screening for DVT was not performed in patients in our study. Thus, the 16% rate of VTE complications in our study, including COVID-19 patients hospitalized in non-ICU and not undergoing routine ultrasound screening, may indicate a higher VTE risk in our cohort compared to the studies of Porfidia et al. [4] (9%) and Nopp et al. [8] (8%). In addition, in the latter, an incidence of PE and DVT in the non-ICU group was 3.5% and 4.1%, respectively, which were lower rates compared to our study. Perhaps, this was due to the fact that a significant portion of the COVID-19 population included in our study, though not being hospitalized in the ICU, had a more severe course of COVID-19 as evidenced by a frequent need for receiving oxygen therapy including HFNC and ventilation therapy. The large number of patients with a severe course of COVID-19 requiring hospitalization and the inability to provide all patients requiring HFNC and a ventilator with a bed in an ICU during the COVID-19 pandemic in Poland led to a situation when some patients requiring ICU treatment were hospitalized in non-ICUs.

The existing evidence indicates that hospitalized patients with severe COVID-19 are at high VTE risk despite prophylactic anticoagulation [5,28,29]. A meta-analysis by Kollias et al. [28] revealed that the incidence of PE and DVT among patients with COVID-19 who were screened/assessed for VTE (lower limb ultrasonography for DVT and CTA for PE) was 32% and 27%, respectively, with an increasing trend among patients admitted to ICU, most of whom received thromboprophylaxis. Thus, VTE prevalence was high and appeared to be higher in studies with <50% of patients anticoagulated [28]. In another study [27], involving 208 COVID-19 patients hospitalized in a non-ICU who received thromboprophylaxis (patients receiving anticoagulant treatment were excluded from the study), a total of 18% were diagnosed with VTE, of which 8% were PE. These results are consistent with our findings as far as the rate of VTE diagnosis is concerned; however, our study included not only patients receiving thromboprophylaxis but also those receiving anticoagulant treatment. In our study, according to the national guidelines on managing patients with COVID-19, all patients received thromboprophylaxis (83.4% of entire cohort) unless they required anticoagulant treatment for various reasons (16.6%) [15]. Based on our findings, among patients on anticoagulant treatment, 16% developed VTE, and among patients receiving thromboprophylaxis, 15.9% developed VTE. This may indicate that anticoagulant treatment did not protect against the development of VTE in patients with COVID-19 with elevated D-dimer levels hospitalized in the non-ICU. However, we did not observe that patients receiving thromboprophylaxis were at a higher risk of developing VTE than those treated with anticoagulants. Similar results were observed in a multicenter study conducted by Sholzberg et al. [32], who concluded that the administration of therapeutic heparin compared to thromboprophylaxis had no effect on either the development of VTE or the primary composite outcome of death, non-invasive or invasive mechanical ventilation, or ICU admission in moderately ill patients with COVID-19 and increased D-dimer levels admitted to hospital. However, albeit not significant, a noticeable reduction in mortality and a low risk of bleeding was observed with therapeutic heparin in that study [32]. Also, in the study by Avruscio et al. [5], including patients with COVID-19 admitted to both medical wards and ICU, who underwent systematic screening for VTE during their hospital stay, a high frequency of VTE (27% and 76%, respectively) was observed, despite standard-dose or high-dose thromboprophylaxis in all patients. The optimal dose of anticoagulant prophylaxis in patients with COVID-19, which would be safe and ensure the lowest rate of hemorrhagic complications, that affect approximately 2–7% of patients, is currently being discussed [33,34,35].

Our findings indicate that the need for oxygen therapy in patients with COVID-19 is associated with an increased risk of VTE. Among the COVID-19 patients included in our analysis, oxygen therapy in various forms was provided in a total of 60% of patients, of which 72% had developed VTE and 58% had not. Oxygen therapy was given depending on the patient’s requirements using different methods of oxygen supply, such as nasal cannula, oxygen face mask, HFNC, or ventilator. Although SpO_2_ results were not included in our analysis, it is known that oxygen therapy is given to patients with hypoxia diagnosed on the basis of lowered SpO_2_ and arterial blood oxygen levels. Based on our analysis results, there was an increased risk of VTE in COVID-19 patients who required receiving oxygen therapy. However, once patients were divided into various types of oxygen therapy–passive (nasal cannula, oxygen face mask), HFNC, or ventilator—it was not confirmed that specific types of oxygen therapy were significantly associated with the development of VTE complications. While no significant differences between the VTE and non-VTE groups in the types of administered oxygen therapy were observed, patients with VTE complications were most likely to require oxygen therapy, especially HFNC. The lack of statistical significance may be due to the small group of examined patients with VTE complications in each of the subgroups. The effect of hypoxia on the development of VTE complications has been reported in studies showing that those living in a mountainous region are more likely to develop VTE complications than those living at lower altitudes [36,37]. Thachil [38] speculates that hypoxia may be one of the risk factors for increased VTE complications among patients with COVID-19. Lee Yi et al. [3] also report that in their study, as in ours, patients with VTE complications were more likely to require oxygen therapy. Another explanation may be that the need for oxygen therapy resulted from a more severe course of COVID-19, e.g., in bedridden patients, which may also be associated with a higher risk of VTE complications.

We observed that D-dimer plasma levels, which are related to both coagulation and fibrinolysis, were elevated during hospitalization for COVID-19 in both the VTE group and the non-VTE group, and more so for the VTE group. On admission, whereas patients from the non-VTE group had 2-fold higher D-dimer than the reference value, D-dimer in patients from the VTE group was 3-fold higher. While no significant difference was found between the groups on hospital admission, in our study, the maximum level of D-dimer was significantly higher in patients with VTE compared to those without. Numerous other studies showed that the course of COVID-19 is associated with an increase in D-dimer levels as a result of coagulopathy; however, there is a disagreement about the cut-off value of D-dimer that could serve as a predictor of VTE [5,6,8,10,29,35]. Also, a significant association between VTE risk and the D-dimer peak level was found [29]. In the study of Avruscio et al. [5], increased D-dimer levels significantly correlated with a risk of VTE and death. Our findings suggest that higher D-dimer levels on hospital admission may indicate an increased risk of VTE during hospitalization; however, more evidence is needed to confirm this observation. Nevertheless, it should be noted that although our patients were not screened for PE and DVT, we monitored D-dimer levels very closely throughout their stay and each sudden elevation in D-dimers was followed by an adequate imaging diagnostics including CTA of the chest to exclude PE. This approach may be crucial for the early diagnosis of PE. The fact is that the clinical diagnosis of PE was extremely difficult because most COVID-19 patients developed dyspnea due to pneumonia, which is also the most common symptom of PE.

There is a limited amount of data in the literature which analyzed various aspects of VTE in COVID-19 patients infected with various SARS-CoV-2 variants. This may be due to a lack of standardized testing for SARS-CoV-2 variants, as it was the case in our study. Based on the national database data on the SARS-CoV-2 variants [13], we assumed that the Delta variant (B.1.617) predominated in Poland from 1 June to 31 December 2021 and the Omicron variant (B.1.1.529) predominated from 1 January to 30 April 2022. As a result, the patient population analyzed in our study included 110 patients with the Omicron variant infection and 72 patients with the Delta variant infection. In our observation, VTE occurred more frequently in patients with a Delta variant infection (19.4%) compared to those with the Omicron variant infection (13.6%); however, the difference between the groups was not statistically significant. Based on our observations, we found that patients infected in the later period of our study, when the Omicron variant was predominant, had a significantly milder course of COVID-19 compared to those with the Delta variant infection. This is particularly interesting as Grobbelaar et al. [1] observed that hypercoagulability in Omicron-infected patients is less intensive compared to Delta-infected ones. In contrast to our findings, Arabi et al. [39] reported a higher risk of VTE in patients with the Omicron variant infection than the Delta variant infection, but the difference between groups was not significant.

Our work aimed to present real-world clinical data. Our single-center study included the analysis of a comprehensive set of various data that were retrospectively collected, a thorough review of medical records, well-defined study outcomes, and a relatively large sample size. Regarding the limitations, it should be noted that the further generalization of our findings requires caution associated with the use of selected variables which were analyzed with the univariate regression analysis. The effect of pneumonia severity on the development of VTE complications and that of VTE occurrence on the risk of death and transfer to the ICU were not analyzed. Also, it would be desirable to perform the genomic sequencing of the genetic code of SARS-CoV-2 to determine the specific variant that caused COVID-19.

## 5. Conclusions

The risk of VTE including PE and DVT in patients hospitalized in the non-ICU due to Delta and Omicron SARS-CoV-2 variant infections is high despite thromboprophylaxis or anticoagulant treatment. Hospitalized COVID-19 patients with VTE had significantly higher D-dimer plasma levels compared to patients without VTE. Continuous D-dimer monitoring during hospitalization is required for the early detection of VTE. A diagnosis of arterial hypertension and ongoing oxygen therapy are associated with an increased risk of developing VTE during hospitalization for COVID-19.

Our findings may help to identify patients hospitalized for COVID-19 who are at high risk of VTE and, ultimately, contribute to improving patients’ outcomes. Further research is needed to investigate the individualized VTE risk in patients with COVID-19 and determine the optimal preventive therapies.

## Figures and Tables

**Table 1:** Clinical characteristics of the group with (VTE group) and without (non-VTE group) VTE complications among patients hospitalized for COVID-19 in the non-ICU.

Variable	VTE Group *n* = 29	Non-VTE Group *n* = 152	*p*-Value between Groups VTE and Non-VTE
Age (years)	71 (±15)	69 (±16)	0.9
Gender: female/male *n* (%)	13/16 (44/56)	67/85 (44/56)	0.97
Arterial hypertension *n* (%)	22 (75.9)	90 (59)	0.14
Diabetes *n* (%)	8 (27.5)	55 (36.2)	0.15
Coronary artery disease *n* (%)	9 (31)	38 (25)	0.39
Chronic heart failure *n* (%)	5 (17.2)	33 (21.7)	0.14
Atrial fibrillation *n* (%)	7 (24.1)	28 (18.4)	0.36
Cancer *n* (%)	3 (10.3)	34 (22.3)	0.18
History of VTE *n* (%)	5 (17.2%)	0	0.15
Chronic kidney disease (≥stage 3) *n* (%)	6 (20.7)	27 (17.8)	0.9
Chronic pulmonary disease *n* (%)	4 (13.8)	14 (9.2)	0.18
Smoking *n* (%)	6 (20.7)	31 (20.4)	0.82
Neurological disorders *n* (%)	8 (27.5)	28 (18.5)	0.18
Obesity *n* (%)	7 (24.1)	31 (20.4)	0.82
Omicron SARS-CoV-2 variant *n* (%)	15 (51.7)	95 (62.5)	0.49
Delta SARS-CoV-2 variant *n* (%)	14 (48.3)	57 (37.5)	0.49
Oxygen therapy *n* (%)	21 (72.4)	88 (57.9)	0.32
Anticoagulant treatment *n* (%)	5 (17.2)	25 (16.4)	0.79
Thromboprophylaxis *n* (%)	24 (82.7)	127 (85.5)	0.71

For most parameters, data represent the number of patients (*n*) including the percentage of the total number (in parenthesis). For age, the mean value with standard deviation (in parenthesis) is given. Abbreviations: non-ICU—non-Intensive Care Unit; SARS-CoV-2—Severe Acute Respiratory Syndrome Coronavirus 2; VTE—venous thromboembolism.

**Table 2:** D-dimer plasma levels in the group with (VTE group) and without (non-VTE group) VTE complications among the COVID-19 patients hospitalized in the non-ICU.

Variable	VTE Group *n* = 29	Non-VTE Group *n* = 152	*p*-Value between Groups VTE and Non-VTE
D-dimer on admission (ng/mL)	1549 (766, 4929)	1111 (664, 2050)	0.09
Maximum D-dimer (ng/mL)	5724 (1940, 17,307)	2200 (1086, 4434)	0.005

Abbreviations: COVID-19—Coronavirus Disease 2019; non-ICU—non-Intensive Care Unit; VTE—venous thromboembolism; for D-dimer levels, median values with lower and upper quartiles (in parenthesis) are given.

**Table 3:** Risk of VTE complications in patients hospitalized for COVID-19 in the non-ICU in a univariate analysis.

Variable	Pooled OR for VTE	*p*-Value
Arterial hypertension	2.59	<0.05
History of VTE	2.55	0.12
Oxygen therapy	2.43	<0.05
Neurological disorders	1.58	0.33
Delta SARS-CoV-2 variant	1.55	0.28
Chronic pulmonary disease	1.54	0.49
Atrial fibrillation	1.41	0.47
Thromboprophylaxis	1.35	0.60
Chronic kidney disease	1.13	0.81
Obesity	1.10	0.84
Anticoagulant treatment	1.06	0.92
Chronic heart failure	0.90	0.85
Smoking	0.89	0.83
Omicron SARS-CoV-2 variant	0.64	0.28
Diabetes	0.57	0.25
Cancer	0.33	0.10

Abbreviations: COVID-19—Coronavirus Disease 2019; non-ICU—non-Intensive Care Unit; OR—odds ratio; SARS-CoV-2—Severe Acute Respiratory Syndrome Coronavirus 2; VTE—venous thromboembolism. Results are presented according to decreasing values of odds ratios.

**Table 4:** Risk of VTE complications in patients hospitalized for COVID-19 in the non-ICU depending on the type of oxygen therapy.

Type of Oxygen Therapy	VTE Group *n* = 29	Non-VTE Group *n* = 152	*p*-Value	Pooled OR for VTE
Passive oxygen therapy *n* (%)	13 (44.8)	58 (38.1)	0.50	1.32
HFNC oxygen therapy *n* (%)	2 (6.9)	5 (3.2)	0.11	3.34
Mechanical ventilation *n* (%)	6 (20.7)	25 (16.4)	0.33	1.6

Abbreviations: COVID-19—Coronavirus Disease 2019; HFNC—high-flow nasal cannula oxygen; OR—odds ratio; SARS-CoV-2—Severe Acute Respiratory Syndrome Coronavirus 2; VTE—venous thromboembolism.

## Data Availability

The data presented in this study are available on request from the corresponding author. The data are not publicly available due to privacy restrictions.
